# Supplementary material for: WordCommentsAnalyzer: A windows software tool for qualitative research
Source: F1000Res. 2018 Sep 4;7:536. Originally published 2018 May 3. [Version 2] doi: 10.12688/f1000research.14819.2 (PMC6137411; doi:10.12688/f1000research.14819.2)
Supplement: Supplementary file 3 [file f1000research-7-17614-s0002.tgz › b2a79496-4ada-47c4-9682-a99ce867836d.zip!/Eight Iranian Health Journals/Archives of Rehabilitation_56.docx]

© Archives of Rehabilitation. This work is licensed under CC BY-NC 4.0 (https://creativecommons.org/licenses/by-nc/4.0/).

Volume & Issue: دوره 6 - شماره 4

Title: ارزیابی ارتزهای بلند اندام تحتانی به‌لحاظ اصول بیو مکانیکی، بالینی و فنی‌مهندسی

http://rehabilitationj.uswr.ac.ir/article-1-64-fa.html

هدف: هدف کلی از این پژوهش، تعیین میزات تطابق ارتُز بلند اندام تحتانی (Long Leg Brace) ساخته شده، با استاندارد‌های مورد قبول دانش ارتوپدی فنی بوده است. این استانداردها عبارتند از اصول بیومکانیکی مطرح مانند محل محور حرکتی مفاصل زانو و مچ پا، اصول ارتوپدی فنی (دستور العمل طراحی و ساخت) و اصول بالینی (چگونگی تماس و قرار گیری ارتُز روی اندام) همچنین مقایسه میزان رعایت موارد فوق بین مراکز دولتی و خصوصی شهر تهران سال 1382. روش بررسی: مطالعه حاضر به صورت توصیفی-تحلیلی-مقطعی انجام گردیده و در آن، بریس بلند اندام 193 نفر که با روش تصادفی طبقه ای انتخاب شده‌اند توسط یک پرسشنامه تعریف شده مورد ارزیابی قرار گرفتند. یافته‌ها: با بررسی هفت متغیر در گروه اصول بیومکانیک مشخص شد که در %86/28 از موارد بریسهای ساخته شده، عدم تطابق با اصول تعریف شده وجود داشته است. در گروه سوالات بالینی نُه متغیر بررسی شدند و در %30 از موارد عدم تطابق با اصول مشخصه بالینی در بریسهای اندام تحتانی مشاهده گردید. با بررسی اصول ارتوپدی فنی توسط 23 متغیر، ملاحظه گردید در %25/4 از موارد بریسهای ساخته شده به این لحاظ ایراد داشتند وجود داشت. نتیجه‌گیری: ضمن مطالعه دریافتیم که بریسهای ساخته شده دارای عیوبی هستند و در مواردی که قابل توجه نیز هست با معیارهای مورد قبول طراحی و ساخت مطابقت ندارند و مشکلاتی را برای فرد دریافت کننده و بخصوص هنگام فعالیت پیش می‌آورند.

Title: بررسی مقایسـه‌ای اثـر کشش به روش‌هـای هارمونیک و پی.ان.اف

http://rehabilitationj.uswr.ac.ir/article-1-65-fa.html

هدف: افزایش انعطاف پذیری و کاهش سفتی عضله، از دیدگاه متخصصین توانبخشی و طب ورزشی اهمیت زیادی دارد. در فیزیوتراپی و تربیت بدنی، برای مقابله با کوتاهی عضلات روش های متعددی وجود دارد. هدف این مطالعه، مقایسه اثر کشش به روش های پی ان اف و هارمونیک بر انعطاف پذیری عضله چهار سرران است. روش بررسی: این پژوهش، به روش مداخله ای تجربی (کار آزمایی بالینی تصادفی) انجام شده است. تعداد 45 نفر دانشجو در محدوده سنی 18 تا 35 سال، به روش نمونه گیری آسان (Sample of Convenience) انتخاب شدند و در سه گروه 15 نفرۀ هارمونیک، پی.ان.اف و کنترل قرار گرفتند. آزمودنی ها بدون اختلالات دردناک کمر و اندام تحتانی در شش ماه قبل از شرکت در تحقیق بودند، عضله چهار سرران آنها نسبتا کوتاه بود (20 درجه کمبود در تست اکستانسیون فعال زانو AKE) و سابقه فعالیت ورزشی حرفه ای نداشتند. متغیرهای مورد بررسی عبارتند از سفتی عضله و طول همسترینگ که زاویۀ پوپلیته در تست AKE شاخص آن است. در یک مطالعۀ مقدماتی، تکرار پذیری اندازه گیری این دو متغیر مورد تأیید قرار گرفت و سپس افراد گروه هارمونیک به روش هارمونیک و افراد گروه پی ان اف به روش پی ان اف به مدت 6 هفته تحت تمرینات کششی قرار گرفتند،رژیم تمرین 5دقیقه در روز و سه روز در هفته بود. در مورد گروه کنترل اقدام خاصی صورت نگرفت. یافته‌ها: در این مطالعه مشخص شد که طول همسترینگ در اثر هر دو روش کشش، دارای تغییرات معنی داری بود (در هر دو مورد P>0/001) ولی تغییرات گروه کنترل معنی‌دار نبود. تفاوت بین میزان تغییرات ناشی از دو روش کشش، از نظر آماری معنی دار نبود. اندازه سفتی عضله فقط در گروه هارمونیک دارای تغییر معنی دار بود (P>0/03). نتیجه‌گیری: یافته‌های این تحقیق نشان می‌دهند که هردو روش هارمونیک و پی ان اف به یک اندازه در افزایش طول همسترینگ مؤثر هستند، و روش هارمونیک می‌تواند به عنوان جایگزین روش های دیگر کشش مورد استفاده قرار بگیرد. شاید روش هارمونیک بهتر از روش پی ان اف باشد، زیرا همان گونه که ذکر شد اندازه سفتی فقط در این گروه تغییرات معنی‌داری داشته است؛ یعنی نه تنها طول عضله افزایش می‌یابد بلکه تغییرات چشمگیر بیومکانیکی در عضله در اثر این روش ایجاد می‌شود که نمود این تغییرات کاهش سفتی در این گروه است؛ و شاید افزایش طول در گروه پی. ان. اف تغییرات واقعی در انعطاف پذیری نبوده و فقط تحمل افراد به کشش زیاد شده است.

Title: نگرش دانشجویان خوابگاهی دانشگاه علوم پزشکی ایران در مورد رفتارهای انحرافی درخوابگاه‌ها - تهران 1384

http://rehabilitationj.uswr.ac.ir/article-1-67-fa.html

هدف: محیطهای بسته خوابگاهها و مراکز جمعی، محیطهای مناسبی برای بروز رفتارهای انحرافی است. دراین تحقیق نگرش دانشجویان درمورد رفتارهای انحرافی درخوابگاههای دانشجویی مورد بررسی قرار گرفته است. روش بررسی: ازجمع تعداد 2014 نفر دانشجویان پسرودختر مقیم در10خوابگاه ، تعداد 325 نفر بصورت نمونه گیری تصادفی (138 نفر پسر و 187 نفر دختر) انتخاب و ابتدا با دانشجویان خوابگاهها در محل هرخوابگاه، مصاحبه متمرکز گروهی (F.G.D) با شرکت تعداد حداکثر 15 نفر دانشجو در هر جلسه و بوسیله محققین همکار، انجام و سپس با تهیه پرسشنامه مستند به داوری اهل نظر ونتایج حاصل از مصاحبه‌ها، اطلاعات ازدانشجویان ازطریق تکمیل پرسشنامه اخذ گردید. این پژوهش توصیفی است و بدلیل توصیفی بودن فاقد فرضیه است و یافته های پژوهش پاسخ به هفت سئوال مطرح شده برای محقق است. یافته‌ها: یافته‌های پژوهش، حاکی براین است که عواملی ازقبیل نحوه توزیع در اتاقها، کاهش شرم حضورناشی ازکمبود فضای تعویض لباس وسوسه های جنسی دررفتار انحرافی درخوابگاه موثر بوده وعامل غیرمذهبی بودن و آشفتگی خانواده را، زمینه ساز رفتار انحرافی درخوابگاه ها دانسته و از نظر جنسیت، دانشجویان پسر، بیش از دانشجویان دختر، خودارضایی و در مقابل دختران، بیش از پسران، نیازهای جنسی خود را سرکوب نموده‌اند. نتیجه‌گیری: ‌بین شرایط خوابگاهی و شرایط خانوادگی و شرایط جامعه و رفتار انحرافی در خوابگاههای دانشجویی رابطه وجوددارد.

Title: تأثیر تحریک تالاموس بر پردازش‌های زبانی و حافظه در بیماران پارکینسون

http://rehabilitationj.uswr.ac.ir/article-1-69-fa.html

هدف: تحریک عمقی هسته VL تالاموس به عنوان روش درمانی موفقی برای بعضی اختلالات حرکتی در بیماران پارکینسونی مقاوم به دارو گزارش شده است. این امر مشارکت مدارهای قشری- زیر قشری را در کنترل حرکت تایید می‌کند. در خصوص مشارکت این مدارها در کارکردهای شناختی و بویژه زبان تحقیقات کمی انجام شده و اختلاف نظر وجود دارد. هدف این پژوهش تعیین میزان اثر تحریک عمقی تالاموس چپ و راست، بر پردازشهای زبانی و حافظه می‌باشد. روش بررسی: تحقیق حاضر از نوع چند مورد پژوهی است که طی آن پردازشهای زبانی و حافظه6 بیمار پارکینسونی مقاوم به دارو پس از عمل جراحی تحریک عمقی (Deep Brain Stimulation) هسته VL تالاموس (دو بیمار تالاموس راست، دو بیمار تالاموس چپ، دو بیمار دوطرفه)، در دو وضعیت دستگاه خاموش و روشن با حداقل فاصله زمانی 40 روز بین دو آزمون، مورد بررسی و مقایسه قرار گرفتند. ابزار تحقیق و روش جمع‌آوری داده ها شامل پرسشنامه (تاریخچه بیماری)، آزمون حافظه وکسلر، زیر آزمون درک دستوری و جمله‌سازی آزمون BAT) Bilingual Aphasia Test) و روانی کلامی بود. یافته‌ها: براساس نتایج آزمونها، نمره آزمون حافظه وکسلر در گروه تالاموس چپ در حالت تحریک در مقایسه با عدم تحریک افزایش جزئی و در گروه تالاموس راست کاهش جزئی نشان داد. نمره درک جملات پیچیده در هر دو گروه، در حالت تحریک تالاموس در مقایسه با وضعیت عدم تحریک افزایش نشان داد. در نمره روانی کلامی و جمله سازی در حالت تحریک تالاموس در مقایسه باحالت عدم تحریک در هر دو گروه شاهد کاهش نمره بودیم. نتیجه‌گیری: با توجه به یافته‌های این پژوهش به نظر می‌رسد، تحریک عمقی تالاموس باعث بهبود مهارت درک دستوری و کاهش روانی کلامی و ساخت جمله در هر دو گروه شده است که از نظریه مشارکت دوطرفه تالاموس در پردازشهای زبانی حمایت می‌کند و مغایر با تحقیقاتی است که این تغییر را فقط در اثر تحریک تالاموس چپ گزارش کرده اند. به نظر می رسد تفاوت اثر تحریک عمقی تالاموس چپ نسبت به راست در نمره حافظه تاییدی بر سوبرتری تالاموس چپ باشد .

Title: درمان و نتایج ترمیم آسیب‌های اسپاگتی مچ

http://rehabilitationj.uswr.ac.ir/article-1-70-fa.html

هدف: هدف از مطالعه حاضر بررسی نتایج حاصل ترمیم در ضایعات (Spaghetti Wrist) می‌باشد. از آنجا که این گونه آسیب‌ها اعصاب و تاندونها را تواماً درگیر می‌کند، اختلالات عملکردی زیادی را بر جا می‌گذارد و نیاز به بررسی و مداخلات درمانی مناسب دارد. روش بررسی: مداخله حاضر روی 33 بیمار ضایعه فوق که در طی سال‌های 84-1383 به مرکز دست ایران مراجعه کرده بودند انجام شد. روش درمان در همه یکسان بود. به این صورت که مداخله زودهنگام حسی و حرکتی انجام شد. در نهایت بیماران تحت ارزیابی حسی و حرکتی قرار گرفتند و نتایج بازگشت حسی و حرکتی پس از 10 هفته بین دو عصب اولنار و مدین درگیر بررسی شد. همچنین تأثیر سایر عوامل مداخله‌گر مثل زمان جراحی، زمان ارجاع، سن و جنس بر نتایج حاصله ارزیابی شد. یافته‌ها: 33 بیمار در این مطالعه تحت بررسی قرار گرفتند. در بررسی حسی عصب مدین در هیچکدام از موارد بی حسی کامل دیده نشد و 37/40 درصد بیماران کاهش لمس سبک را داشتند. اما در بررسی حس عصب اولنار 7/43 درصد نفر بی‌حسی کامل و 25/56درصد نفر کاهش حس حفاظتی داشتند. همه افراد فلکشن گپ صفر داشتند. بنابراین مشکل حرکتی و لغزش تاندون نداشتند. از نظر قدرت، درگیری توأم عصب اولنار و مدین بیشترین تأثیر را روی کاهش قدرت داشت. متغیر سن به دلیل نزدیک بودن دامنه سنی بیماران اثری روی نتایج نداشته وتفاوت میزان قدرت و Pinch با 0=P و ضریب همبستگی 0/99 ارتباط مستقیم داشتند. نتیجه‌گیری: ترمیم اولیه و زودهنگام و مداخله درمانی سریع نتایج عملکردی خوبی را در این ضایعات به دنبال خواهد داشت. تحریک حس لمس و شروع تمرینات بازآموزی از مراحل اول درمان، بهبود حس و بازگشت حس را در اعصابی که آسیب دیده‌اند بهبود می‌بخشد.

Title: بررسی تأثیر مدل مراقبت پیگیر بر کنترل وزن و فشار خون بیماران همودیالیزی

http://rehabilitationj.uswr.ac.ir/article-1-71-fa.html

هدف: علت عمده بستری شدن بیماران همودیالیزی در بیمارستان افرایش فشار خون و بار مایعات بدن می‌باشد. این مطالعه با هدف تعیین تاثیر مدل مراقبت پی‌گیر بر میزان فشار خون و کنترل وزن بیماران همودیالیزی در سال 84-1383 در شهر همدان انجام شده است. روش بررسی: این پژوهش یک مطالعه نیمه‌تجربی از نوع طرح اندازه‌های تکراری می‌باشد. نمونه‌های این پژوهش را 38 بیمار مبتلا به بیماری مرحله انتهایی کلیه تشکیل می‌داد که با توجه به شرایط و روش نمونه‌گیری تصادفی در یک گروه در مدت 6 ماه مورد بررسی قرار گرفتند. ابزار جمع‌آوری اطلاعات شامل پرسشنامه‌ دموگرافیک و چک‌لیست کنترلی بود. پژوهش‌گر در مرحله اول پژوهش، مرحله آشناسازی را انجام داد و تا انتهای دوره تنها پرسشنامه‌ها و چک‌لیست‌های کنترلی تکمیل شد. پس از اتمام مرحله اول پژوهش، بلافاصله پژوهشگر اقدام به اجرای مداخله در مرحله دوم پژوهش بر روی همان گروه نمود. سپس با استفاده از نسخه 13 نرم‌افزار آماری SPSS، آمار توصیفی و آزمون آماری آنالیز واریانس در اندازه‌گیری‌های مکرر مورد بررسی و تجزیه و تحلیل قرار گرفت. یافته‌ها: در تجزیه و تحلیل داده‌ها، آزمون آماری آنالیز واریانس در اندازه‌گیری‌های مکرر نشان داد که بین میانگین سطوح فشارخون سیستولیک و دیاستولیک در شش بار اندازه‌گیری قبل و بعد از اجرای مدل تفاوت معنی‌داری وجود دارد (P<0/05). همچنین آزمون آماری آنالیز واریانس در اندازه‌گیری‌های مکرر بین زمان‌های مختلف اندازه‌گیری وزن تفاوت معنی‌داری را نشان نمی‌دهد. نتیجه‌گیری: با توجه به یافته‌های پژوهش می‌توان نتیجه گرفت که با اجرای مدل مراقبت پی‌گیر تفاوت معنی‌داری در میزان فشارخون بیماران همودیالیزی ایجاد می‌شود.

Title: توان‌بخشی اسکیزوفرنیا: در انتها یا از آغاز؟

http://rehabilitationj.uswr.ac.ir/article-1-72-fa.html

بخش عمده بیماران روانی که نیازمند بستری طولانی مدت و توانبخشی هستند را مبتلایان به اسکیزوفرنیا تشکیل می‌دهند. قسمت زیادی از این بیماران همراه با بیماری خود به سنین سالمندی رسیده اند و علاوه بر عوارض بیماری اسکیزوفرنیا با کمبودها و بیماریهای ناشی از سالمندی هم روبرو هستند. برخلاف دهه های گذشته که درمان مؤثری برای بیماری اسکیزوفرنیا وجود نداشت، امروزه می‌توان با تشخیص فوری و درمان بهنگام بیماری با داروهای مؤثر از ازمان و مقاومت به درمان جلوگیری کرد و ضمن بهبود چشمگیر پیش آگهی بر کیفیت زندگی بیماران و مراقبان آنان افزود. در حال حاضر تقسیم بندی مصنوعی بیماران اسکیزوفرنیک به انواع حاد و مزمن اعتباری ندارد و در عین حال توصیه می‌شود به جای برپا کردن بیمارستانهای ویژه بیماران روانی که موجب جدا افتادن بیماران از اجتماع می گردد در بیمارستانهای عمومی بخش‌هایی به این بیماران اختصاص داده شود تا ضمن برخورداری از مراقبتهای درمانی و توانبخشی بهتر از عواقب روانشناختی و اجتماعی بستری در بیمارستانهای اختصاصی روانی هم محفوظ بمانند.

Title: آکرودیس‌اوستوزیس: گزارش یک جوان مبتلای 21 ساله ایرانی

http://rehabilitationj.uswr.ac.ir/article-1-74-fa.html

آکرودیس استوزیس یکی از بیماریهای نادر مادرزادی است. کمتر از 80 مورد تابحال در نشریات پزشکی گزارش شده است. اغلب موارد گزارش شده تک مورد در خانواده بوده اند. اما ابتلای یکی از والدین و فرزندان، همچنین سن بالای پدر در بعضی خانواده هامشاهده شده که احتمال وراثت غالب اتوزومی را مطرح می نماید. نشانه های بالینی بقدر کافی مشخص است، و پزشکان باتجربه براحتی می توانند بیماری را تشخیص دهند. در این مقاله یک جوان مذکر 21 ساله ایرانی گزارش می‌شود که یافته های بالینی دروی تمام عیاربا تشخیص آکرودیس اوستوزیس همخوانی دارد. بر اساس تجربه ماوپیگیری های صورت گرفته، احتمالااین اولین مورد بیماری گزارش شده در ایران می‌باشد. مسلماً موارد دیگری هم باید در جمعیت عقب مانده کشورمان باشند که تشخیص آنها معلوم نشده است. بنابراین همکاران محترم در برخورد با بیماران عقب مانده ذهنی دیس مورفیک باید به نشانه های این بیماری توجه نمایند تا آنرا به درستی تشخیص بدهند. تشخیص بیماری در واقع کلینیکی ورادیولوژیکی است و نیازی به بررسی های آزمایشگاهی پیچیده ندارد.
